# Numerical analysis of in vivo platelet consumption data from ITP patients

**DOI:** 10.1186/s12878-015-0034-4

**Published:** 2015-10-19

**Authors:** Ted S. Strom

**Affiliations:** Department of Pathology and Laboratory Medicine, Memphis Veterans Administration Medical Center, 1030 Jefferson Ave, Memphis, TN 38104 USA; Department of Pathology and Laboratory Medicine, University of Tennessee Health Sciences Center, Memphis, TN USA

**Keywords:** Platelets, Thrombocytopenia, Immune thrombocytopenic purpura, Numerical analysis

## Abstract

**Background:**

Numerical methods have recently allowed quantitative interpretation of in vivo murine platelet consumption data in terms of values for the random destruction rate constant (RD), intrinsic lifespan (LS), and the standard deviation of ln LS (SD), as well as the platelet production rate (PR) and age distribution (AD). But application of these methods to data obtained in thrombocytopenic patients is problematic for two reasons. First, such data has in all cases been obtained with radiolabeled platelets, and uptake of the radio-isotope by long lived cells complicates the analysis. Second, inferred values of the platelet production rate (PR) and random destruction rate (RD) are difficult to interpret, since increased RD can occur either as a cause or a consequence of thrombocytopenia.

**Methods:**

We used a numerical method to analyze in vivo platelet consumption data from a series of *41* patients with immune thrombocytopenic purpura (ITP). An additional parameter, the fraction of labeled long-lived cells (LL), was evaluated concurrently with RD, LS, and SD. To provide a basis for interpreting these values, we used an iterative interpolation process to predict their response to different pathophysiologic mechanisms. The process also generates predicted effects on the widely used immature platelet fraction (IPF).

**Results:**

Optimal parameter value sets were identified in 76 % (31 of 41) of the data sets. 27 of 31 ITP patients showed no substantial homeostatic increase in platelet production, with the remaining 4 showing both augmented platelet consumption and a compensatory increase in PR. Up to 1/3 of the patients showed the degree of increased RD expected to result from reduced thrombopoiesis only. “Jacknife” resampling yielded CV values of <0.5 in over 75 % of the evaluable data sets. Predicted platelet age distributions indicate that interpretation of the IPF and absolute IPF (aIPF) is a complex function of platelet count. We found, counter-intuitively, that reduced PR can *increase* the IPF, and increased RD can *reduce* the aIPF.

**Conclusions:**

Our findings support the feasibility of using numerical analysis to quantitatively interpret in vivo platelet consumption data, to identify likely etiologies of thrombocytopenias, and to assess the utility of IPF measurements in that context.

**Electronic supplementary material:**

The online version of this article (doi:10.1186/s12878-015-0034-4) contains supplementary material, which is available to authorized users.

## Background

In vivo platelet consumption studies have often been used to quantify the rates of both random and lifespan-dependent consumption processes, and to evaluate production rate, but their interpretation can be problematic. The aim of the present study is to demonstrate that a numerical analysis method can reliably quantify these rates, and thereby identify fundamental pathophysiologic features, in thrombocytopenic patients.

Methods which have been used to interpret such studies include a simple exponential decay model [1], a weighted mean method that applies an empirical mixture of separately optimized linear and exponential decay processes [2], a purely lifespan-dependent model [3], the Mills-Dornhorst equation (which includes but does not solve for a random (exponential) consumption rate constant) [4, 5], the widely used multiple hit model (based on a unique consumption mechanism for which there is little experimental support) [6, 7], and combined use of the latter two approaches [8, 9].

None of these methods allow concurrent modeling of the random (hemostatic and phagocyte-mediated) and lifespan-dependent processes known to result in most in vivo platelet consumption. Numerical analysis models have been designed for that purpose, and their utility for the analysis of murine platelet consumption data has been demonstrated [10, 11].

It is not clear, however, whether these methods can be adapted to evaluate existing clinical data, since the latter in all cases involves tracking of radiolabeled platelets and includes contributions from uptake of the radio-isotope by other, longer-lived cell types. It is also unclear how to translate the resultant kinetic parameter values into useful conclusions about how individual patients became thrombocytopenic. Here we have modified a previously described numerical model [10] to successfully analyze and interpret published data from a series of patients with immune thrombocytopenic purpura (ITP) [12].

## Methods

### Patients

Entry criteria for the ITP patients have been described previously [12]. The study, including informed consent procedures, was performed according to the principles outlined in the Declaration of Helsinki of 1975. Briefly, ^111^Indium-labeled autologous platelet consumption data from 41 consecutive adult patients with prednisone non-responsive primary ITP was reviewed. The data was obtained either (A) at the time of diagnosis, or (B) after failure to sustain a platelet count response to prednisone treatment. Diagnostic criteria for all patients included exclusion of other malignant, metabolic, or pharmacologic causes, as well as causes of “secondary” ITP such as hepatitis C virus (HCV) infection. For those in group (A), rapid consumption of autologous ^111^Indium-labeled platelets (interpreted via the multiple hit model [6]) was an additional diagnostic criterion. For those in group (B), demonstration of antiplatelet antibodies on the platelet surface via indirect immunofluorescence [13] was used for this purpose.

All of the patients in this study underwent splenectomy after failing to respond adequately to prednisone treatment. Patients were deemed to have had a complete response to splenectomy if their platelet count persistently exceeded 100 × 10^9^/L thereafter, with no significant bleeding episodes. They were considered “non-responders” if their subsequent platelet count did not exceed either 30 × 10^9^/L, or twice their baseline count, or if they had persistent significant bleeding episodes.

### Platelet kinetics studies

These have been described in detail previously [12]. Briefly, platelet rich plasma was prepared by differential centrifugation, and platelets prepared by a subsequent high speed centrifugation were labeled with ^111^Indium oxine by standard methods. Peripheral blood specimens obtained at 30 min after injection were considered “baseline” measurements (for patient 30, a 1.5 h time point was used), and all subsequent measurements were normalized to these for each patient. Equilibration with a pool of splenic platelets is thought to be complete well within this initial time frame [14]. All post-injection specimens for each patient were evaluated in a gamma counter at the same time to eliminate decay-related effects on recovery.

### Numerical analysis

Data analysis was performed on desktop computers using Microsoft Excel. Baseline (initial) parameter ranges searched were RD 0–19 (resolution 1 %) %/h, LS 0–15.2 % (resolution 0.8 %), and SD 20–267 h (resolution 13 h). LL and RD ranges and resolutions were then empirically optimized to the values shown in Table 1. The equilibration metric was calculated as the net platelet count produced by the model at the midpoint of the equilibration phase divided by the net platelet count at the end of the equilibration phase, as described previously [10]. All searches achieved an equilibration metric value of > 0.997 (1000 interval equilibration phase, 0.5 h per interval). Searches of resampled data sets were performed over the same parameter ranges shown in Table 1. For cases in which all resampled data sets yielded the same parameter values as the complete data set at resolution “R”, the upper limit of the standard deviation was estimated by the value obtained had one resampled data set yielded a parameter value one “R” range removed from that of the complete data set.

**Table 1:** Patient characteristics, search parameter ranges, and optimal parameter values

Patient characteristics		Search parameter ranges			Optimal parameter values and residuals (SS/n)								
Patient	Platelets (x 10e9/L)	n (data points)	RD (%/h)	LL (%)	RD (%/h)	CV	LS (hr)	CV	LL (%)	CV	SD (of ln LS)	PR (K/ul/h)	SS/n
1	30	6	0–4.75	0–15.2	2.5	0.08	189	0.39	6.4	0.44	0.2	0.77	0.43
2	119	9	0–4.75	0–15.2	0.75	0.39	228	0.2	12.8	0.3	0.2	0.89	6..92
3	165	9	0–4.75	0–15.2	2	0.15	176	0.2	8.8	0.11	0.3	3.55	1.81
4	24	9	4.5–9.25	0–15.2	6.75	0.13	267	na*	4.8	0.31	0.2	1.62	8.26
5	80	9	3.5–8.25	0–15.2	5.5	0.06	215	na*	4.8	0.23	0.2	4.4	2.61
7	41	9	1.0–5.75	0–15.2	3.5	0.06	176	0.51	6.4	< 0.13**	0.1	1.44	12
8	58	8	0–4.75	15.2–30.4	2	0.43	202	0.36	23.2	0.44	0.1	1.64	42.5
11	10	5	9.5–14.75	0–15.2	12.5	0.02	267	na*	7.2	1.81	0.2	1.25	19.3
13	44	6	0–4.75	0–15.2	1.5	0.28	189	0.39	9.6	0.63	0.1	0.71	15.8
14	17	5	9.5–15.25	0–15.2	12.25	0.11	72	2.94	0	(sd < 0.06**)	0.3	2.08	7.02
17	8	9	0–4.75	0–15.2	1.75	0.2	215	0.56	10.4	1.05	0.1	0.14	36.3
18	88	7	0–4.75	0–15.2	2	0.2	150	0.77	8	0.34	0.2	1.92	3.3
20	45	9	2.5–7.25	0–15.2	5.25	0.11	228	0.4	6.4	0.42	0.2	2.36	22.1
21	143	9	0–4.75	8.8–24.0	0	(sd < 0.22**)	124	< 0.12**	11.2	0.16	0.2	1.34	5.82
22	85	8	0–4.75	0–15.2	1.25	0.32	111	0.14	5.6	0.43	0.3	1.61	1.49
23	119	9	0–4.75	15.2–30.4	0	(sd = 0.22)	124	0.09	22.4	0.07	0.3	1.2	4.03
24	20	6	0–4.75	0–15.2	2.5	0.8	33	0.66	1.6	0.42	0.3	1.04	21.4
25	39	9	2.0–6.75	0–15.2	4.5	0.09	228	0.75	4.8	0.2	0.2	1.76	8.2
27	2	9	5.5–10.25	0–15.2	8.25	0.06	176	0.73	11.2	0.18	0.1	0.17	4.77
28	22	9	0–4.75	0–15.2	2.25	0.2	254	0.35	13.6	0.25	0.1	0.5	24.4
29	34	9	2.0–6.75	0–15.2	4.5	0.23	189	0.85	6.4	0.4	0.1	1.53	18.1
30	13	6	0–4.75	0–15.2	1.75	0.24	137	0.25	6.4	1.05	0.2	0.26	6.28
32	36	5	3.0–7.75	0–15.2	7.25	0.04	72	0.53	2.4	0.27	0.1	2.63	0.38
33	238	7	0–4.75	13.6–28.8	0.25	2.56	163	0.47	20.8	0.39	0.2	2.33	10.2
34	77	6	2.0–6.75	16.8–32.0	4.5	0.27	124	0.35	24	0.23	0.1	3.49	5.51
35	37	9	0.5–5.25	0–15.2	3	0.48	137	1.02	5.6	0.36	0.3	1.17	13.4
36	32	6	4.5–9.25	0–15.2	5.75	0.15	111	0.58	6.4	0.95	0.2	1.85	5.88
37	102	6	0–4.75	11.2–26.4	3	0.58	228	0.31	17.6	0.43	0.1	3.07	49.5
38	170	7	0–4.75	17.6–32.8	0.25	1.2	150	0.07	24.8	0.05	0.1	1.45	2.81
39	21	6	3.0–7.75	0–15.2	5.25	0.35	111	1.27	12.8	0.39	0.2	1.12	27.3
40	43	6	2.5–7.25	0–15.2	5	0.35	124	na*	14.4	0.41	0.2	2.16	15.8
Normalized	191	23	0.05–1.0	na	0.5		140		na		0.2	2.12	57.3

Platelet counts were obtained at the time of the study. Patients 1,2, and 3 showed a major subsequent response to splenectomy (see text). Resolution is equal to 5 % of the search ranges shown. The “*normalized”* data set is pooled data from the three patients (3, 33, and 38) whose platelet counts transiently exceeded 150 K/ul in response to prednisone. CV values were obtained by “jackknife” resampling (see text). “na*” denotes cases for which one or more of the resampled or complete data sets yielded LS values at the high end of the search range. Values marked by ** were for cases in which all of the resampled data sets yielded the same optimal parameter value (see Methods)

## Results and discussion

### Modeling in vivo platelet turnover

The numerical model used here [10] posits that an in vivo platelet population can be visualized in a spreadsheet as a series of small platelet cohort concentrations. The cohorts are assumed to be produced at a constant rate (PR, K/ul/h) in short sequential time periods, and individually consumed, by both random and lifespan-dependent processes, at the end of each such time period. The consumption curve for individual cohorts is determined by a random destruction rate constant (RD, %/h), by the lognormally distributed cohort lifespan (LS, hr), and by the standard deviation of ln LS (SD). Population platelet consumption curves are generated by summing the cohort values at sequential time points. Optimal theoretical consumption curves generated by a large range of possible parameter values are identified via quantitative comparison to each data set (summed squared residual values, or SS).

For the 41 patient studies analyzed here, each patient’s platelet consumption data was normalized to the first (baseline) measurement of circulating ^111^In. Visual inspection of the data strongly suggests that many of the labeled platelet preparations contained long lived species, as others have described in similar studies [15, 16]. This is evident in the plateau phase seen at late times in the consumption data (for example, patients 35 and 27, Fig. 4). To take this into account, we evaluated a fourth parameter: the fraction of the labeled cells/platelets consisting of long lived (species (LL, %). This parameter simply “shrinks” the scope of the analysis to the consumption of platelets from 100 % of the time zero value to an optimizable minimum percentage (LL). For modeling purposes, lifespans of these long lived species are assumed to be infinite.

### Optimal parameter value search process

**Fig. 1:**
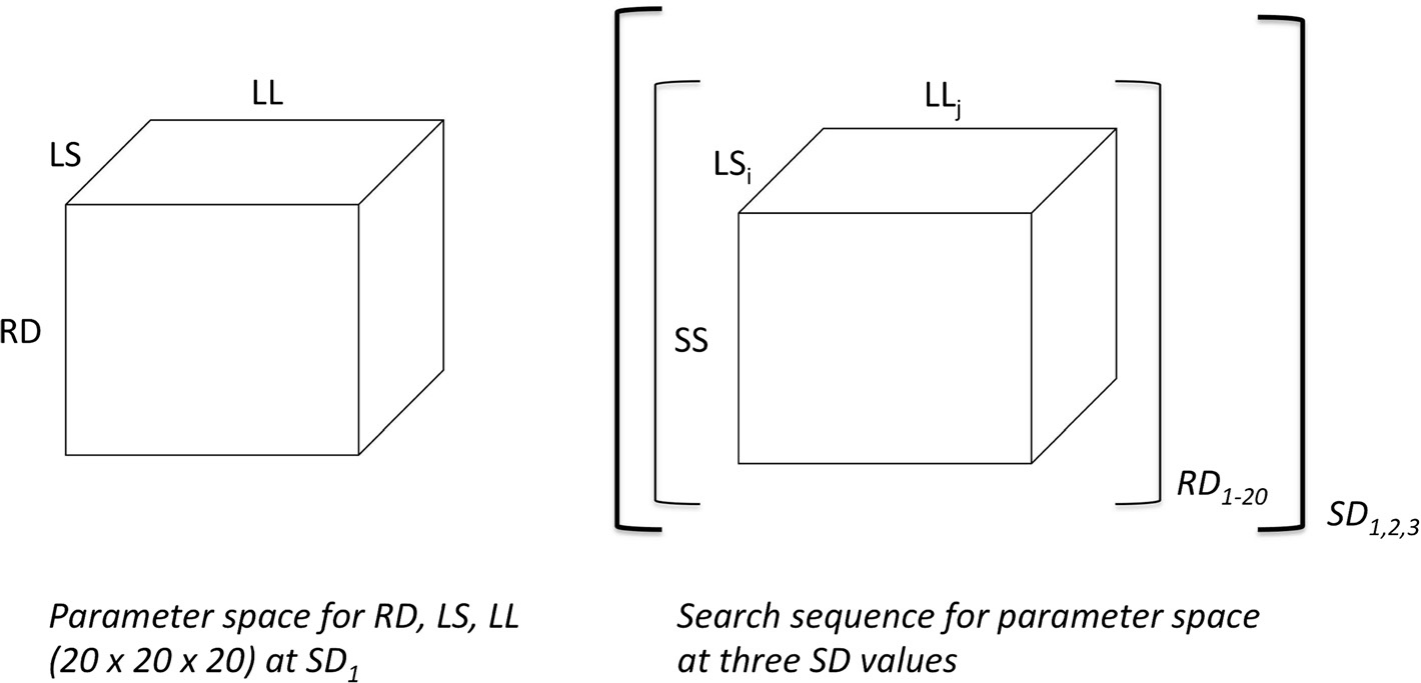
Optimal parameter search schematic. Left: parameter space evaluated for each data set. Right: Schematic of search sequence used. For each data set, SS values were calculated for each point in each RD-defined plane in parameter space. Minimal SS values were identified for each plane; each plane was evaluated at 20 RD values; and each RD value was evaluated at 3 SD values

**Fig. 2:**
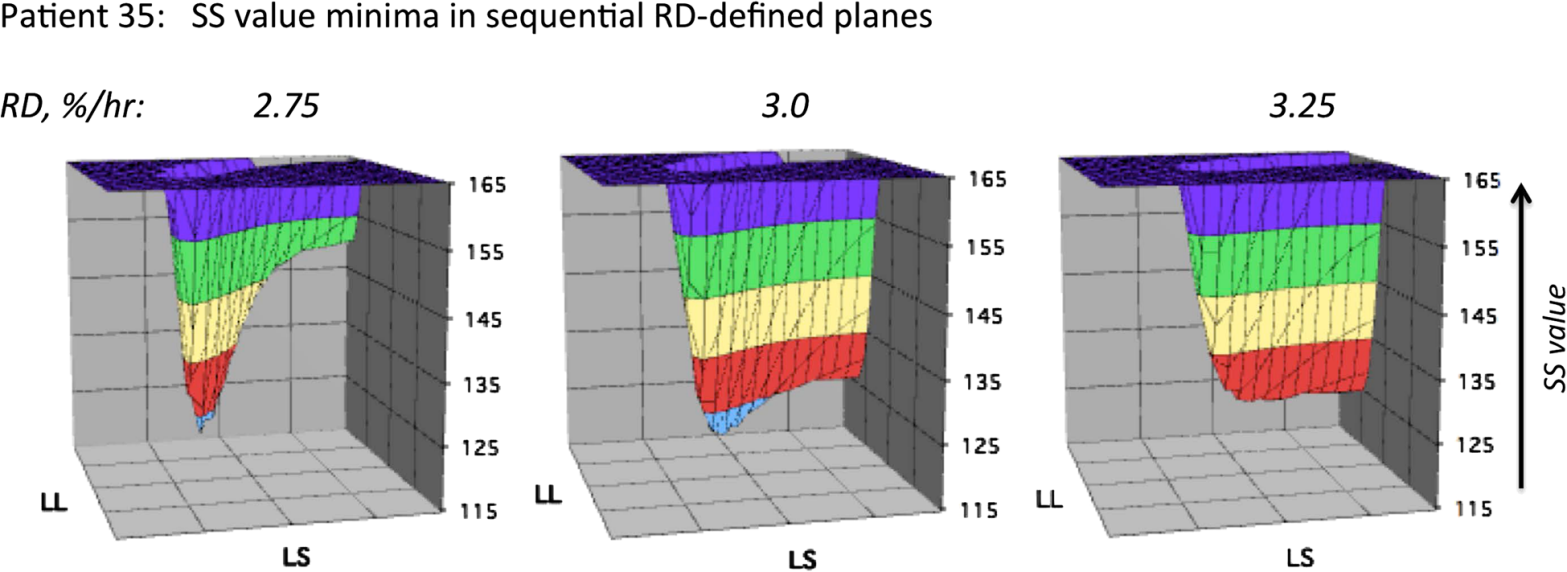
Identifying optimal parameter values. The graphs demonstrate three ‘planar’ SS value minima, of which the minimum in the plane defined by RD = 3.0 %/h is the ‘volume’ minimum at this SD value (0.3). The parameter values associated with this SS minimum yield the consumption curve for this patient shown in Fig. 4. Horizontal axis scales are as follows: For LL, 0 to 15.2 %, resolution 0.8 %. For LS, 20 to 267 h, resolution 13 h. See Table 1 for additional data

**Fig. 3:**
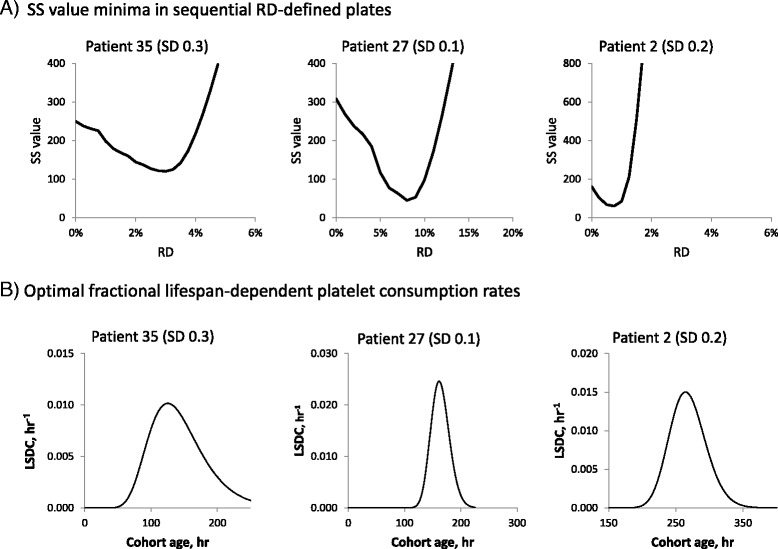
Optimal parameter value searches and lifespan distributions. Top: The examples shown yielded optima in three different SD-defined parameter spaces. The low points on the SS value curves define the optimal parameter value sets. Resolution is 0.25 %/h (patients 35, 2), 1 %/h (patient 27). Bottom: the optimal fractional lifespan dependent consumption rate (LSDC) distributions for these optima are shown. For example, the optimal parameter values for patient 35 demonstrate the distribution of lifespan-dependent consumption rates per cohort (peaking at 1 %/h) shown at left. Resolution is 0.5 hr

**Fig. 4:**
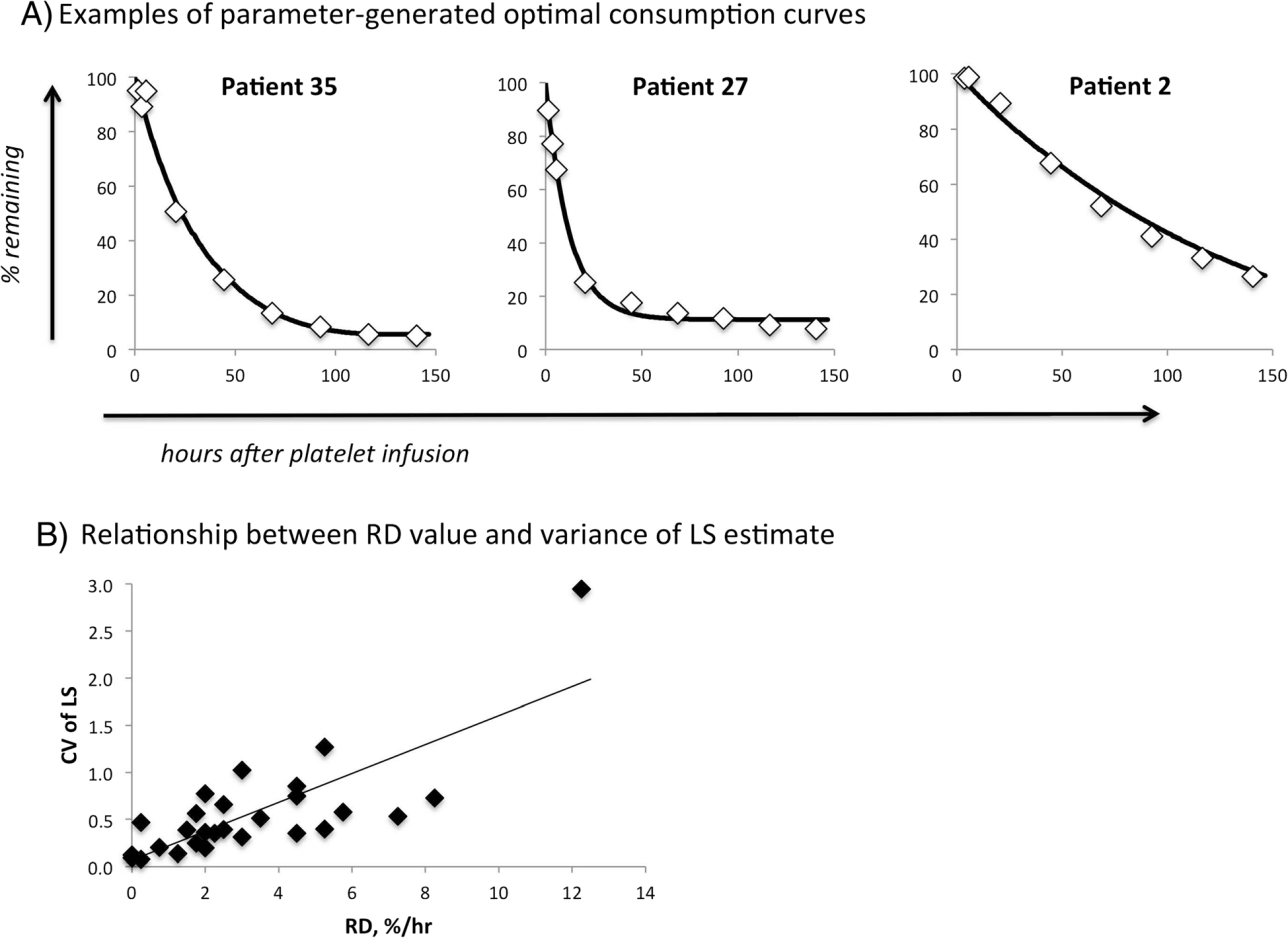
Modeled consumption curves, LS variance. **a** Examples of parameter-generated optimal modeled consumption curves. The data points shown for each patient were used to infer optimal parameter values (RD, LS, SD, and LL; Table 1) predictive of the consumption curves shown. **b** Relationship between RD value and variance of LS. Values are from Table 1

Optimal parameter value searches were performed as shown schematically in Fig. 1. For a given data set, SS values are determined for each possible set of parameter values in a four-dimensional parameter space defined by RD, LS, SD, and LL. The core component of the search is an evaluation of SS for each point in a 20 × 20 plane of possible LS and LL values at fixed values of RD and SS. The resultant minimum “planar” SS values are visually identifiable (see examples in Fig. 2). This process is repeated over a range of 20 RD values, yielding in most cases single “volume” SS minima as shown in Fig. 3a. Finally, the entire process is repeated at a series of SD values, and the resultant volume minima are compared in order to identify a “global” minimum SS value and its associated parameter values. Distinguishable alternative volume minima showing SS values greater than those of the global minima were also seen in some data sets (see below). Searches were performed for only three SD values, as this generated a plausible range of distribution widths for the resultant lifespan-dependent consumption rates (see examples in Fig. 3b) while significantly reducing computation time. Examples of the consumption curves generated by the optimal parameter values are shown in Fig. 4.

### Data quality evaluation and optimal parameter value search results

**Fig. 5:**
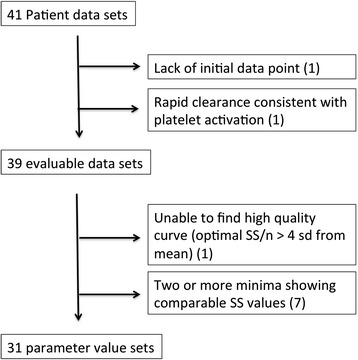
Data quality evaluation. Schematic showing criteria used to remove non-evaluable data sets from evaluation. The first data point obtained for the patient lacking an initial data point was obtained at 6 h after infusion of labeled platelets

This process is outlined in Fig. 5. Of the 41 originally reported ITP patient data sets, one was excluded due to lack of initial time point data. One patient demonstrated an initial platelet clearance rate of 46 % in the first 1.5 h of the study (>4 standard deviations faster than the mean). Because this value suggests the type of platelet activation during labeling/processing that we have on occasion seen in murine platelet clearance studies (TS, unpublished), this data was also excluded. For the remainder, quality of parameter value optimization was evaluated in terms of the ratio of SS to n (the number of data points per patient data set), where n ranged from 5 to 9 (Table 1). One case (patient 31) with an SS/n value of 143 (over four standard deviations from the mean value of 18.5) was then excluded. No other SS/n values fell beyond two sd from the mean.

A single “global” minimum SS value, with its associated (optimal) parameter values, was identified in 24 of the 39 evaluable data sets. The optimal consumption curves show a large amount of inter-patient variation, as the examples in Fig. 4a demonstrate. Of those showing more than one minimum, convincing global minima were identified in four data sets on the basis of goodness-of-fit. Specifically, the global minima in these cases showed SS values which were less than 50 % of those defining the alternative (local) minima. Three data sets showed global minima for which comparison of absolute vs. squared residuals provided additional support for their significance (see Additional file 1). Seven data sets, however, showed local minima that could not be distinguished from the global minima on these bases. In sum, we were able to identify convincing global minima in 31 of the 39 evaluable data sets (79 %) shown in Table 1.

Data quality was further evaluated by performing “jackknife” resampling studies on each of the patient data sets in Table 1 [17]. Optimal parameter values were obtained for each of the n subsets for each data set via the same process used to analyze each complete data set (at the SD value of the complete data set’s global minimum). We found CV values for calculated RD and LL parameters to be under 0.5 for over 75 % of these cases (Table 1). Quantification of platelet lifespan was more difficult, with only 52 % of our cases showing CV values for the LS parameter of under 0.5. That is expected, however, because LS value estimates showed a larger variance in cases where random destruction predominated (Fig. 4b).

### Predicting the effects of reduced platelet production and increased random destruction

As a guide to interpreting the parameter values in Table 1, we used the model to predict how a normal platelet population’s consumption parameter values might shift in response to A) impaired production, B) increased consumption, or C) increased consumption in association with a homeostatic increase in platelet production. Our assumptions were:

i) The optimal parameter values (RD_0_, LS_0_, and SD_0_) and the associated platelet production rate (PR_0_) obtained for the three patients in the study whose platelet counts transiently normalized in response to prednisone (patients 3, 33, and 38, Table 1) are representative of normal.
ii) RD is comprised of two component processes: Hemostatic RD (HRD) and non-hemostatic RD (NHRD) (i.e. RD = HRD + NHRD). Substantial hepatic NHRD is a well characterized phenomenon [18].
iii) The absolute HRD value at a normal platelet count (aHRD_0_) makes up a given normal fraction (“f”) of absolute RD (i.e. aHRD_0_/RD_0_ = f). We do not know the normal value of f.
iv) aHRD_0_ is maintained, as platelet count declines, via an increase in HRD and a resultant increase in RD, as suggested by earlier studies of platelet turnover [8].
v) LS is not affected by reduced platelet production. Studies of the genetic basis of platelet lifespan support this assumption [3].

**Fig. 6:**
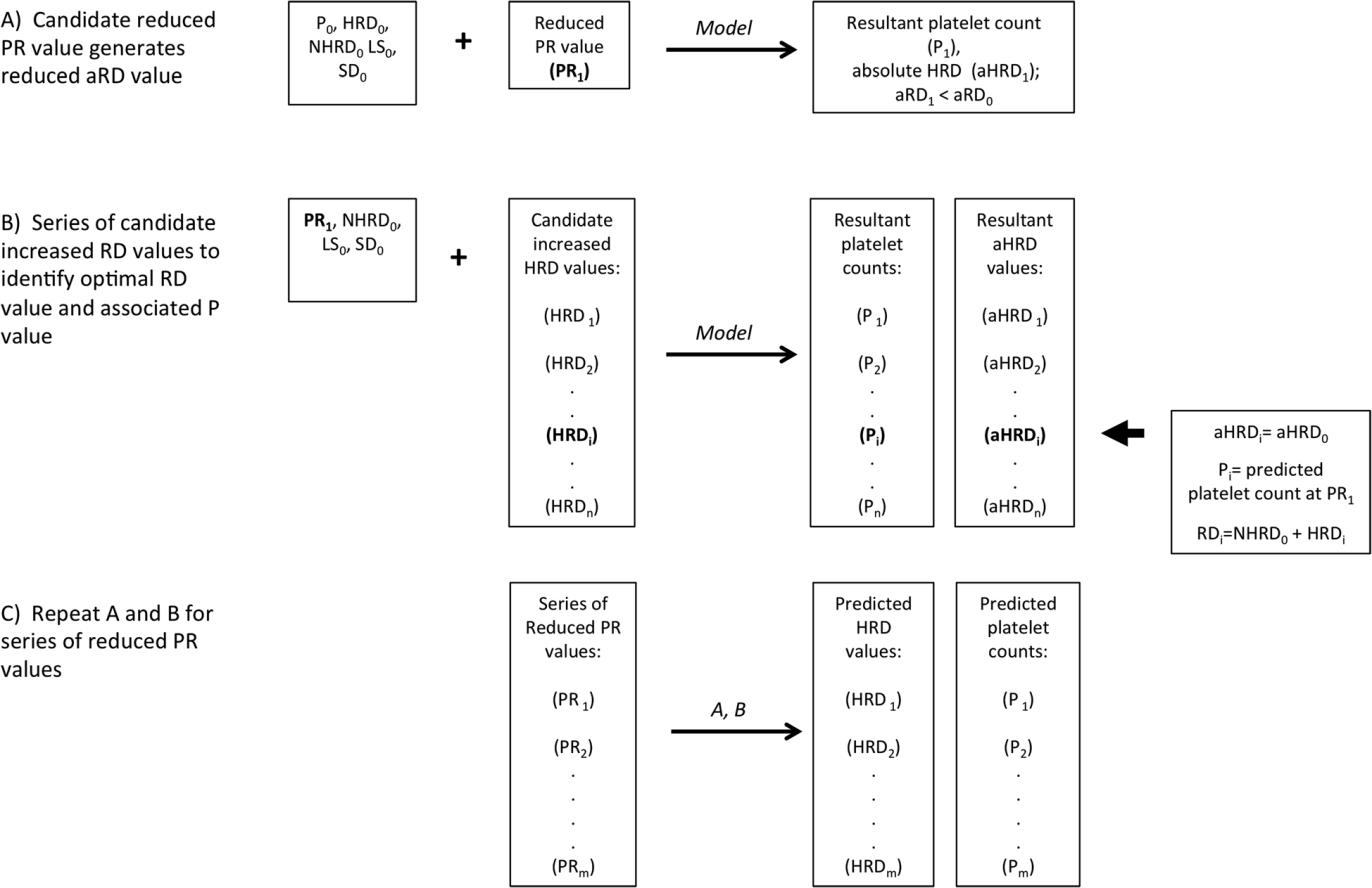
Modeling the effect of reduced Production rate on HRD and Platelet count. Schematic of the interpolation process described in the text

The effect of reduced platelet production (PR) on RD and platelet count was modeled as shown in Fig. 6. The process begins (step A) with the optimal (baseline) parameter values for pooled data from the three patients who transiently normalized their platelet counts (Table 1), using an initial “f” value of 1.0. From this set, a “target” reduced platelet production rate (PR_1_) is generated, corresponding to 90 % of PR_0_. Using that value, the model generates the expected (reduced) aHRD value (aHRD_1_). We then (step B) incrementally increase HRD until the model generated value of aHRD (aHRD_i_) = aHRD_0_. The associated RD_i_ value (=HRD_i_ + NHRD_0_) and platelet count values are those predicted to occur at PR_1_. Finally (step C), we repeat steps A and B with a series of reduced platelet production rates (PR_i_). This generates predicted HRD and platelet count values for each PR_i_ value. We then repeated this analysis at f values of 0.5 and 0.2.

We note that for our baseline parameter values, aRD_0_ (RD × platelet count) is equal to 45 % of PR_0_. We make no quantitative predictions for the effect of reducing PR below aRD_0_ because the assumptions underlying the current numerical analysis model may not hold in that case. This is because the number of hemostatic targets is expected to increase below platelet counts at which hemostasis begins to be impaired. That in turn would invalidate the assumption of a constant absolute HRD rate, which is one of the bases for our iterative predictive method (Fig. 5). A model incorporating a dynamic hemostatic target population will be needed to predict platelet consumption rates in these circumstances.

To model the effect of increased random platelet consumption (RD), we generated a series of incrementally reduced target platelet counts (P_i_)(range: 90 % to 10 % of baseline), and to achieve each we incrementally increased RD from its baseline value until the model generated value of P (P_m_) was equal to P_i_. To model the concurrent effects of increased RD and homeostatically increased PR, we used the same series of target platelet counts (P_i_), and for each we increased PR in a manner proportional to the reduction in platelet count (to a maximum of twice the baseline PR value, a conservative theoretical starting point) before, again, empirically identifying RD_i_.

**Fig. 7:**
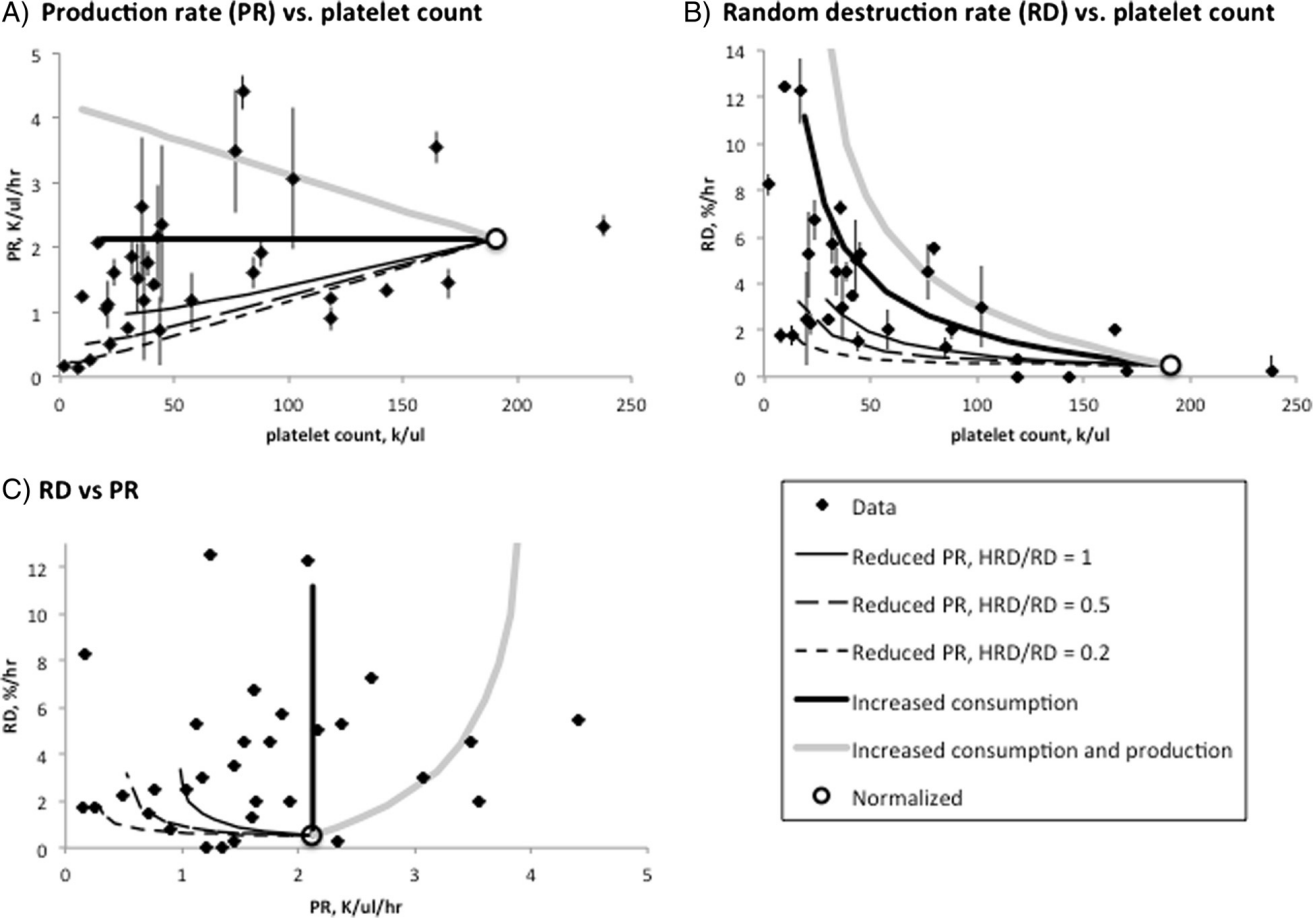
Platelet production and consumption, observed and predicted parameter values. Optimal RD and PR values from Table 1 are plotted for each of the patients in the study. Projected values for thrombocytopenias due to reduced production (at three values of HRD/RD), increased consumption with no homeostatic increase in production, and increased consumption with a compensatory increase in production rate, were interpolated as described in the text. **a** Population turnover rate, which at equilibrium equals platelet production rate, vs. platelet count. **b** Random destruction rate (RD) vs. platelet count. **c** RD and PR values from **a** and **b**. Error bars are standard deviations arrived at via jacknife resampling (see text)

The results of these three modeling approaches are plotted with the values obtained for the patients in Fig. 7.

### Optimal patient parameter values in comparison to modeled values

Surprisingly, only four patients in the study showed a platelet production rate that is even modestly increased (>50 %) in comparison to the presumed normals (Fig. 7c). The latter showed a mean platelet production rate (2.12 K/ul/h) comparable to the 1.7 K/ul/h rate estimated for normals in a previous study [8]. The finding of predominantly low to normal production rates in the thrombocytopenic cases (Fig. 7a) is corroborated by the distribution of random destruction rates (Fig. 7b), where rates consistent with no increase in platelet production are seen for, again, all but a handful of the patients. A surprisingly large number of cases (at least 12 of 31) fall near the RD rates predicted to result solely from impaired platelet production. The predicted rates vary significantly, however, as a function of aHRD_0_/RD_0_ (‘f’).

Because we don’t know the normal value of ‘f’, our ability to predict the increase in RD at low platelet counts is limited. Future studies in patients with thrombocytopenias due to impaired platelet production could resolve that problem.

### Modeling of immature platelet fraction values

An ability to take up fluorescent marker dyes such as thiazole orange (a marker of “reticulated platelets”, RP) or the proprietary dyes used in Sysmex hematology analyzers (marking the “immature platelet fraction”, IPF) is thought to be characteristic of those platelets which have recently been released into the bloodstream. The age threshold (T) at which “young’ platelets stop taking up these marker dyes is not known. Because the numerical analysis model generates a platelet age distribution for any given set of parameter values, it can be used both to estimate T and to predict the effect of altered production and consumption rates on the fraction of platelets of age less than T (i.e. the IPF).

**Fig. 8:**
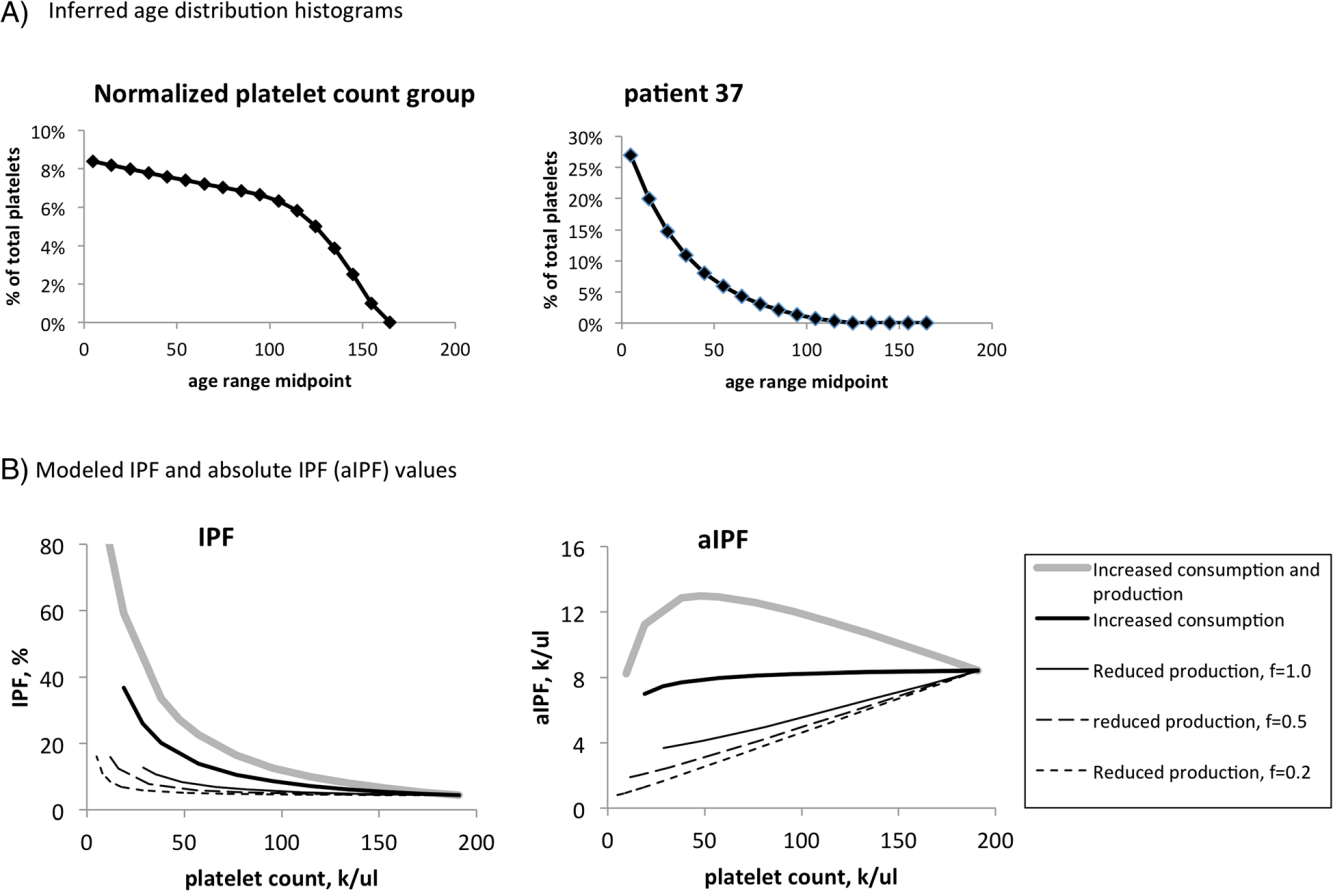
Age distribution histograms and IPF modeling. **a** Histograms were generated from the optimal kinetic parameter values shown in Table 1, using bin sizes of 10 h. **b** Projected IPF and absolute IPF (aIPF) were generated from the parameter values for the normalized platelet count group (Table 1), as described in the text

Specifically, the normal range for the IPF is approximately 4.5 % (each clinical laboratory typically establishes its own range; this is the value in use at the Memphis VA Medical Center). Per the age distribution predicted by the model for our normalized controls (Fig. 8a), the youngest 4.5 % of platelets corresponds to those aged less than 4 h (i.e. T = 4 h). Application of that cutoff to the age distributions generated during modeling of the effects of altered production and/or consumption (Fig. 7), generates the predicted IPF and absolute IPF (aIPF) for thrombocytopenias induced by those mechanisms, as shown in Fig. 8b.

We note that this analysis depends on the assumption that *all* nascent platelets below a given age (T) take up the fluorescent markers used in the RP and IPF assays. Our measurements of mass turnover for mature and reticulated murine platelets suggest that this may not be the case [19].

## Conclusions

Here we have shown the feasibility of quantifying platelet consumption and production rate parameters via numerical analysis of clinical autologous ^111^In-labeled platelet consumption data. Although in some cases more than one set of rate parameters yields a consumption curve that closely fits the in vivo platelet consumption data, we were able to identify a single “global” optimum parameter set for 79 % of the evaluable data sets. We have shown that the technical challenges associated with quantifying the variable amount of long-lived labeled cells in these studies are tractable. It would however be preferable to avoid the need for such calculations via the use of fluorescently labeled, rather than radiolabeled, platelets in this type of study.

Only a small fraction of the prednisone-refractory ITP patients in this study (4 of 39 evaluable patients) showed evidence of a compensatory increase in platelet production rate. Platelet production rates below those of the presumed normals were frequent, and for several patients a thrombocytopenia due solely to impaired platelet production could not be ruled out. More data from normal controls would be needed to confirm these conclusions, as would studies aimed at quantifying the normal (absolute) rate of random hemostatic platelet consumption. Also, it remains possible that a strong homeostatic increase in platelet production rate is characteristic of those ITP patients who demonstrate a durable response to prednisone. If they can be correlated with other types of study (such as evaluation of the immunologic effects on megakaryocyte function that others have reported [20]) [21]), our findings would be consistent with the normal serum thrombopoietin (TPO) levels seen in most ITP patients [22–25]. That observation remains difficult to explain.

Finally, our modeling of the impact of changes in platelet production and consumption rates on the platelet age distribution suggest that there is no simple correlation between the aIPF and the etiology of a given thrombocytopenia. Despite the fact that the platelet count is used to calculate the aIPF, the aIPF can only be interpreted in the context of the platelet count (Fig. 8b). But if the predicted curves in Fig. 8b can be verified by comparison of IPF values to measured clinical platelet production and consumption rates in bone marrow failure patients, the IPF and platelet count could subsequently be used to infer the kinetic bases for most thrombocytopenias.

## Additional file

Additional file 1:
**Optimal parameter search results.** (DOCX 26 kb)

## Abbreviations

RD: Random destruction (%/h)
LS: Intrinsic lifespan (hr)
SD: Standard deviation of ln LS
PR: Production rate (K/ul/h)
AD: Age distribution
ITP: Immune thrombocytopenic purpura
CV: Coefficient of variation (stdev/mean)
IPF: Immature platelet fraction (%)
aIPF: Absolute immature platelet fraction (K/ul/h)
LL: Long lived species (%)
SS: Sum of squared residuals value
SS/n: SS value normalized to the number of data points in a given data set
RD_0,_ LS_0_, SD_0_: Optimal parameter values for the “normalized” subset of patients
HRD: Hemostatic random destruction (%/h)
NHRD: Non-hemostatic random destruction (%/h)
aHRD, aNHRD: Absolute values of HRD and NHRD (K/ul/h)
HRD_0,_ RD_0_: Values of HRD and RD for the “normalized” subset of patients
“f”: Ratio of aHRD_0_ to RD_0_
P_i_: Platelet count at rank “I” in the interpolation process (Fig. 6)
RP: Reticulated platelets
T: Threshold age below which platelets are identified as “immature”
