# Supplementary material for: Numerical analysis of in vivo platelet consumption data from ITP patients
Source: BMC Hematol. 2015 Oct 19;15:14. doi: 10.1186/s12878-015-0034-4 (PMC4615868; doi:10.1186/s12878-015-0034-4)
Supplement: Additional file 1: — Optimal parameter search results. (DOCX 26 kb) [file 12878_2015_34_MOESM1_ESM.docx]

*Optimal parameter search results:*  Squared summed residual values necessarily lend more weight to the initial (higher) values in the consumption curves. For cases in which two or more optima yielded comparable squared residual values, comparison of squared to absolute residual values was in some cases useful. For example:

In this case the local minimum SS value identified an optimal consumption curve which, ‘by eye’, is clearly suboptimal. The squared residual values of the two curves are, however, within less than 16% of each other. Comparison of summed absolute residuals yields the larger fractional difference between the two analyses shown below. Similar results were obtained for patients 4 and 36 (data not shown).

| SS minimum | squared residuals | % of local min | absolute residuals | % of local min |
| --- | --- | --- | --- | --- |
| global | 162.7 | 84.2 | 28.6 | 78.3 |
| local | 193.2 |  | 36.5 |  |
